# Hippocampal formation–cortical high-fidelity memory networks in healthy older adults during the mnemonic discrimination task

**DOI:** 10.3389/fnagi.2026.1788092

**Published:** 2026-07-08

**Authors:** Joseph C. C. Chen, Adam Gazzaley, Peter E. Wais

**Affiliations:** 1 Department of Neurology, Neuroscape, University of California, San Francisco, CA, United States; 2 Departments of Physiology and Psychiatry, University of California, San Francisco, CA, United States

**Keywords:** fMRI, mnemonic discrimination task, functional connectivity, memory, older adults

## Abstract

**Introduction:**

Fidelity of detailed information retrieved from long-term memory (LTM) declines in aging due to changes affecting learning and remembering processes. Such behavioral deficits correspond with functional alterations in the hippocampal formation. However, it is unclear whether age-related decline in high-fidelity retrieval alters functional connections between hippocampal and cortical regions.

**Methods:**

Twenty-two cognitively intact older adults (69.4 ± 4.4 years) completed mnemonic discrimination tasks during fMRI indicating, whether previously encoded targets, lures, or novel objects were old or new.

**Results:**

Participants’ high-fidelity memory, measured by the lure discrimination index (the proportion of “new” responses to lures minus the proportion of “old” responses to novel objects), was 0.45 ± 0.04 – lower than previously reported scores in young adults. High-fidelity LTM in older adults engaged a hippocampal formation region in the left entorhinal cortex with trial-wise beta-series connections to the right angular gyrus and the left precuneus.

**Discussion:**

Taken together, we characterize the distribution of medial temporal lobe (MTL)-cortical networks in older adults that underlies high-fidelity memory.

## 1 Introduction

Healthy older adults often experience declining richness and specificity of details in their long-term memory (LTM). Their declining capabilities to retrieve accurate source, episodic, or contextual information about recent experiences (Chalfonte and Johnson, 1996; Naveh-Benjamin et al., 2003; Stark et al., 2013; Wais et al., 2012) have been attributed to age-related changes in cognitive function that affect both learning (Ferguson et al., 1992) and remembering processes (Hashtroudi and Johnson, 1990). These LTM processes support the flexible connection of various pieces of information related to facts and events remembered in distinct and detailed terms, which we refer to as high-fidelity memory. As the fidelity of memory declines, older adults suffer from increasing retroactive interference and consequently retrieve more false memories (Dodson et al., 2007; Norman and Schacter, 1997).

Functional MRI (fMRI) coupled with memory paradigms has been used to examine the brain regions associated with age-related LTM decline (Angel et al., 2013; Davis et al., 2012; Dulas and Duarte, 2016; Roediger III and Geraci, 2007; Spaniol and Grady, 2012; Velanova et al., 2007; Wang et al., 2016). A key finding, which reflects the necessary role of the medial temporal lobe (MTL) in LTM (Squire and Zola-Morgan, 1991), is that the diminished capability for high-fidelity memory results from age-related alterations in the function of the dentate gyrus and CA3 regions of the hippocampus (Leal and Yassa, 2015; Yassa et al., 2011). Many memory paradigms have confirmed the importance of the MTL in LTM, such as continuous report paradigms, autobiographical retrieval, and mnemonic discrimination tasks. Continuous-report paradigms require participants to report features (such as color or spatial location) along a continuous dimension, and studies have found age-related differences in hippocampal activity during retrieval (Gellersen et al., 2021; Korkki et al., 2023). Autobiographical retrieval paradigms involve participants recalling specific life events during fMRI scanning, which has similarly revealed age-related differences in hippocampal recruitment (Maguire and Frith, 2003; Martinelli et al., 2013; St Jacques et al., 2012). The mnemonic discrimination task (also referred to as the mnemonic similarity task) probes behavior attributed to hippocampus-dependent pattern separation processes to discriminate between previously studied objects and similar lures (Bakker et al., 2008; Stark et al., 2019; Yassa et al., 2011). Taken together, task-based fMRI memory paradigms are promising tools for investigating age-related memory decline.

The present study utilized an object-based mnemonic discrimination task (MDT) to characterize brain regions associated with age-related memory decline. A qualitative decline in older adults’ memory has been linked to a behaviorally observable deficit in mnemonic discrimination (Leal and Yassa, 2015; Stark et al., 2015; Wais et al., 2021; Yassa et al., 2011). Mnemonic discrimination is evidenced by explicit or implicit awareness of subtle changes relative to the memory of a prior experience. Commonly, this awareness helps support the ability to select information from memory that is most relevant for a particular retrieval goal, which yields high-fidelity retrieval of LTM. Previous studies using MDT have shown that hippocampal circuits are crucial for high-fidelity memory retrieval (Bakker et al., 2008), vary with age (Yassa et al., 2011), exhibit hyperactivity in amnestic mild cognitive impairment (aMCI; Yassa et al., 2010), and that reducing this hyperactivity with levetiracetam improves memory in aMCI (Bakker et al., 2012). In looking beyond the hippocampus, increased activation in association with high-fidelity LTM retrieval has been identified in cortical brain regions such as the parahippocampal cortex (Nash et al., 2021), bilateral inferior frontal gyrus (Wais et al., 2017, 2018), bilateral angular gyrus (Wais et al., 2017), and right occipital cortex (Klippenstein et al., 2020) in young adults. Few studies have investigated the broader cortical regions that support mnemonic discrimination in older adults (Mooraj et al., 2025). One study performed a short-delay version of the MDT on 55 healthy older adults in a 7-T MRI and found general linear model (GLM)-based activation across the cortex, including areas such as the left precentral gyrus, left parietal lobe, left supplementary motor area, right lingual gyrus, left middle temporal lobe, right postcentral gyrus, bilateral caudate nucleus, left precuneus, left thalamus, and right amygdala (Yang et al., 2024). To further characterize hippocampal-cortical connectivity, the anatomically defined hippocampal fMRI time series was Pearson-correlated against the rest of the brain, showing significant connectivity between hippocampal ROIs and the frontoparietal network (Yang et al., 2024). However, anatomical ROIs are susceptible to bias, as it is well understood that only a small proportion of voxels are truly active during a particular cognitive event (Poldrack and Mumford, 2009), and GLM-based analyses are inherently averaged across multiple trials. The beta-series functional connectivity metric provides insight by differentiating the relative strength of network signals from the functional MTL seed to cortical regions in association with successful lure rejection versus false alarms.

We aimed to examine the role of functional MTL-lateral cortical networks in the retrieval of high-fidelity memory. By using trial-wise beta series correlation, we build on past studies to investigate MTL–cortical connectivity associated with high-fidelity LTM retrieval in older adults. Although the older adults in our cohort were carefully characterized to meet the age-normal criteria for cognitive performance, we expected that while some participants would retain robust capability for high-fidelity memory comparable to younger adults, others would not (Grady, 2012; Stark et al., 2013; Wais et al., 2021). We aimed to explore whether individual differences in behavioral indices of LTM, relative to MTL activation and associated functional cortical network connectivity, demarcate how successful mnemonic discrimination arises in older adults (Bakker et al., 2012; Cabeza et al., 2018). Indeed, characterization of high-fidelity memory networks in cognitively normal older adults may provide further evidence of network patterns on which older adults, but not young adults, depend. Aging-related accumulations of amyloid plaques in the medial prefrontal and precuneus regions, which have been implicated in changes observed in aging-related cognitive decline (Yassa et al., 2010, 2011), are thought to occur years prior to diagnostic levels of behavioral impairment (Hampel et al., 2021). Aging-related differences in distributed functional networks associated with high-fidelity LTM may therefore represent an additional factor to inform future studies examining correlations across such biomarkers, functional networks, and behavior.

## 2 Methods

### 2.1 Participants and trial design

A total of 23 healthy older adults (mean age: 69.4 *±* 4.4 years, 11 men) participated in this study. The inclusion criteria were as follows: native English speakers, completion of at least 14 years of education, normal or corrected-to-normal vision, no contraindications to MRI, and no use of psychotropic medications. The experimental protocols were performed in accordance with the guidelines, procedures, and regulations approved by the Institutional Review Board (IRB) of the University of California, San Francisco. Participants provided informed consent and received a payment of $50 in compensation for their time. Data from one male participant were excluded from the analysis because of task non-compliance in the scanner.

### 2.2 Neuropsychological testing

All participants underwent 15 standardized neuropsychological tests assessing depression, executive function, and memory, and they were found to be cognitively healthy (within 1.5 standard deviations) relative to normative values for age-matched controls (Table 1). Participants were recruited as older adult controls from a registry of volunteers who had been assessed during a neuropsychological evaluation at the University of California, San Francisco, Alzheimer’s Disease Research Center. The neuropsychological evaluation included the following tests: mini-mental state exam (Folstein et al., 1975), geriatric depression scale (GDS; Yesavage et al., 1982), working memory and verbal learning (California Verbal Learning Test–Second Edition [CVLT-II]; Delis et al., 1987), processing speed (Wechsler Adult Intelligence Scale–Revised [WAIS-R]; Wechsler, 1997), visual-motor sequencing (Delis–Kaplan Executive Function System [DKEFS] Trail-Making A and B), executive function (DKEFS Stroop interference test), and semantic fluency and phonemic fluency (DKEFS; Delis et al., 2001).

**Table 1:** Neuropsychological results.

Neuropsychological test	Mean score (SD)	Normative score
Mini-mental state exam	29.6 (0.7)	29
Geriatric depression scale	3.5 (4.8)	< 9
CVLT: Trial 5 Recall	13.5 (2.2)	10
CVLT: short delay free recall	12.9 (2.6)	7
CVLT: short delay cued recall	13.1 (2.1)	9
CVLT: long delay free recall	12.8 (2.7)	7
CVLT: long delay cued recall	13.2 (2.2)	9
WAIS-R: digit symbol (90s)	60.5 (12.6)	47
DKEFS trial making: numbers (seconds)	36.6 (15.5)	54 < 59
DKEFS trial making: letters (seconds)	36.7 (18.9)	55 < 59
DKEFS stroop: color naming	32.4 (9.2)	33.5
DKEFS stroop: reading words	21.1 (4.7)	25.5
DKEFS stroop: interference	57.3 (13.2)	73
Semantic fluency: animals	21.5 (5.2)	21.7

Mean scores (with standard deviation (SD) in brackets) for standardized neuropsychological tests were used as criteria for normal cognitive performance. Each participant scored within 1.5 SDs of their age-matched normative value for minimum performance. As a reference, the mean of the age-matched normative test values is also shown.

### 2.3 Experimental procedure

This study involving older adults used the same stimuli, behavioral procedures, and neuroimaging protocols as published previously in an fMRI study of younger adults (Wais et al., 2017) based on the mnemonic similarity task (Stark et al., 2013). This study aimed to investigate high-fidelity memory in older adults. This qualitative measure of LTM has been operationalized via the behavioral pattern separation task (Stark et al., 2013), which is hippocampal-dependent (Bakker et al., 2008).

#### 2.3.1 Stimuli

A total of 330 object images were displayed at 1024×768 pixel resolution on an LCD computer monitor using E-Prime 2.0 (Psychology Software Tools, Inc.; Pittsburgh, PA). A total of 140 pairs of images (one target and one similar lure image), along with 50 distinctly novel images, were prepared. Each image presented a common object in color and centered on a white screen. The stimuli were a subset of the Mnemonic Similarity Task (Stark et al., 2013) and were controlled for concreteness and ease of nameability.

#### 2.3.2 Behavioral procedure

**Figure 1:**
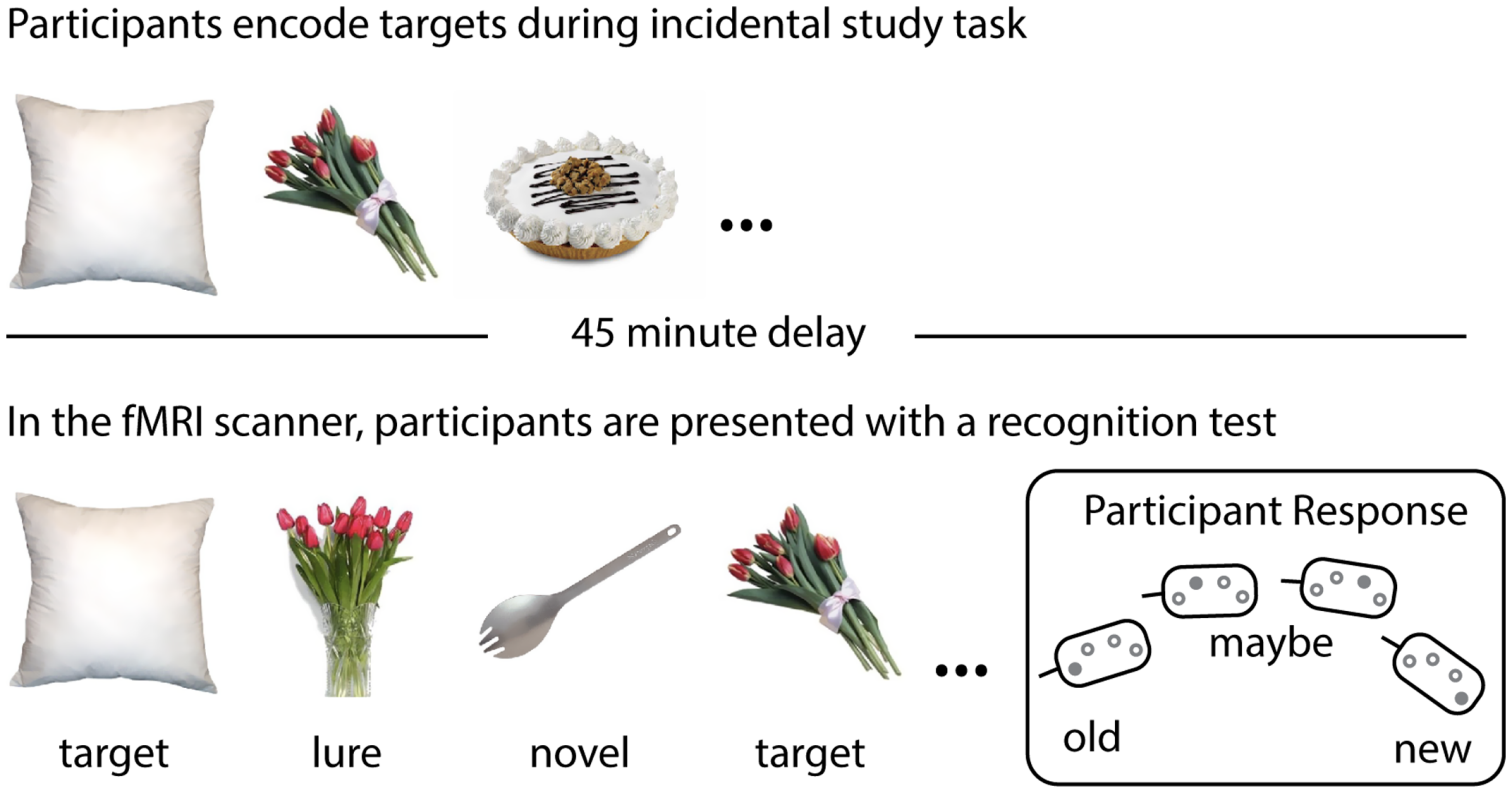
Mnemonic discrimination task paradigm in the functional magnetic resonance imaging (fMRI) scanner.

We presented the task in two sessions with a delay interval of approximately 45 min between the study and test sessions. In the study session, participants viewed one image from each of the 140 object pairs (stimulus presentation was 2.5 s) while responding to two incidental questions designed to promote in-depth visualization of each object: (1) “Yes or no, will the object fit inside a lady’s medium shoebox?” and (2) “yes or no, can you carry the object across the room using only your right hand?” The two questions were presented in separate runs so that the participants viewed each object image (i.e., the target for the test session) twice. Participants were kept naïve to the memory test until they were instructed on the test session before entering the scanner. This paradigm is shown in Figure 1.

The test session involved 10 blocks (corresponding to 10 fMRI scans), each of which presented 33 trials. In each trial, participants viewed one object image on the LCD monitor via a mirror installed on the scanner head coil (stimulus presentation was 2 s). Immediately following the object image, a response screen was presented that cued the participant to enter an old/new recognition rating on the response box held in the right hand, according to a four-level confidence scale. Participants were instructed to indicate whether each item was 1 = definitely old, 2 = maybe old, 3 = maybe new, or 4 = definitely new. A 2 s odd/even numbers task followed to end participants’ engagement in the recognition task before the next trial. Following the odd/even task, a fixation stimulus was presented for 2 to 12 s to jitter the onset for the next trial and optimize signal deconvolution in our rapid-event-related design. Fourteen targets (i.e., studied objects), 14 lures (i.e., very similar objects), and five novel items (i.e., distinctly new objects) were presented in each block for a total run time of 380 s. A target and its paired similar lure were never presented in the same test block, and the order of target first or similar lure first was counterbalanced across all trials. The presentation of the target, lure, and novel trials was randomized within each block, and the block order was counterbalanced across participants.

Verbal instructions and brief practice runs preceded both the study and test sessions, and participants were encouraged to repeat the practice runs until they were comfortable with the timing and procedure of the experimental sessions. Once the participants were comfortably situated in the scanner, they completed one high-resolution structural scan and 10 fMRI runs. This procedure was repeated during the structural scans.

For analysis, trials were later sorted into hits (“old” responses to targets), misses (“new” responses to targets), lure correct rejections (LureCR, “new” responses to lures), lure false alarms (LureFA, “old” responses to lures), novel correct rejections (NovelCR, “new” responses to novel items), and novel false alarms (NovelFA, “old” responses to novel items) based on participants’ responses. While participants could provide “maybe” old/new, their responses were binarized to only old or new in the behavioral and neural analyses.

#### 2.3.3 Behavioral lure discrimination index

Our analysis of high-fidelity memory performance focused on the behavioral measure of each participant’s discrimination of the Lures as new during an old/new recognition task. Correct rejection of Lures as “new” indicated memory judgments driven by underlying discrimination (i.e., any given correct rejection may have identified differences between a Lure and its paired Target or between a Lure and all other objects presented in the procedure). Thus, we used the Lure Discrimination Index (LDI) to assess each participant’s qualitative performance in discriminating lures during the memory test, such that LDI = proportion Lure CR–proportion Novel FA (Wais et al., 2017). This calculation of LDI corrects a participant’s LureCR rate using their NovelFA rate to account for noise, such as guessing.

#### 2.3.4 fMRI acquisition

All images were acquired using a Siemens 3-T Magnetom Trio. The T1-weighted MPRAGE was acquired with repetition time (TR) = 2,300 ms, echo time (TE) = 2.98 ms, flip angle = 9°, inversion time (TI) = 900 ms, 1 mm isotropic voxels, and matrix size = 160 × 240 × 256. Thirty-three 3 mm T2*-weighted gradient-echo slices (no skip, voxel size = 3 mm isotropic, TR = 2000 ms, TE = 28 ms, flip angle = 80°, and 240 mm^2^ field of view) were acquired in an axial oblique orientation parallel to the longitudinal axis of the hippocampus. To achieve 3 mm slices, no skip, and within our desired 2 s repetition time, the field of view resulted in a small sacrifice of whole-brain coverage for some participants. The slice prescription coverage always included the ventral extent of the temporal lobes and, as a compromise, sacrificed a limited area of the superior prefrontal cortex for approximately half of the participants.

### 2.4 fMRI preprocessing and univariate analysis

Functional data were preprocessed using Analysis for Functional Neuroimages (AFNI; Cox and Hyde, 1997) *afni_proc.py,* where slice timing correction, blurring to 4 mm full width at half maximum (FWHM), scaling to 100, and co-registration to the minimum outlier fMRI image were performed. Regression was performed with six motion parameters, third-order polynomial baseline fits, physiological noise regressors extracted from the first three principal components from cerebrospinal fluid (CSF) voxels, AFNI’s “ANATICOR” voxel-wise local white matter nuisance regression (Jo et al., 2010), and a basis “TENTzero” finite impulse response function estimating 0–10 s (6 TRs) of beta coefficients at each Hit, Miss, LureCR, LureFA, NovelCR, and NovelFA condition. To address motion confounds, trials that had excessive motion were handled by not modeling the basis functions and artificially removing the existence of a trial occurring.

For spatial normalization and group analysis, a custom group anatomical template was generated using all of the native-space T1 scans in the cohort with Advanced Neuroimaging Tools (ANTs’) *buildtemplateparallel.sh* (Avants et al., 2011), and a 3 mm template space was generated for group statistical analysis (*3dresample*). Individual statistical data were then transformed into this 3 mm group template space (*antsApplyTransforms*).

**Figure 2:**
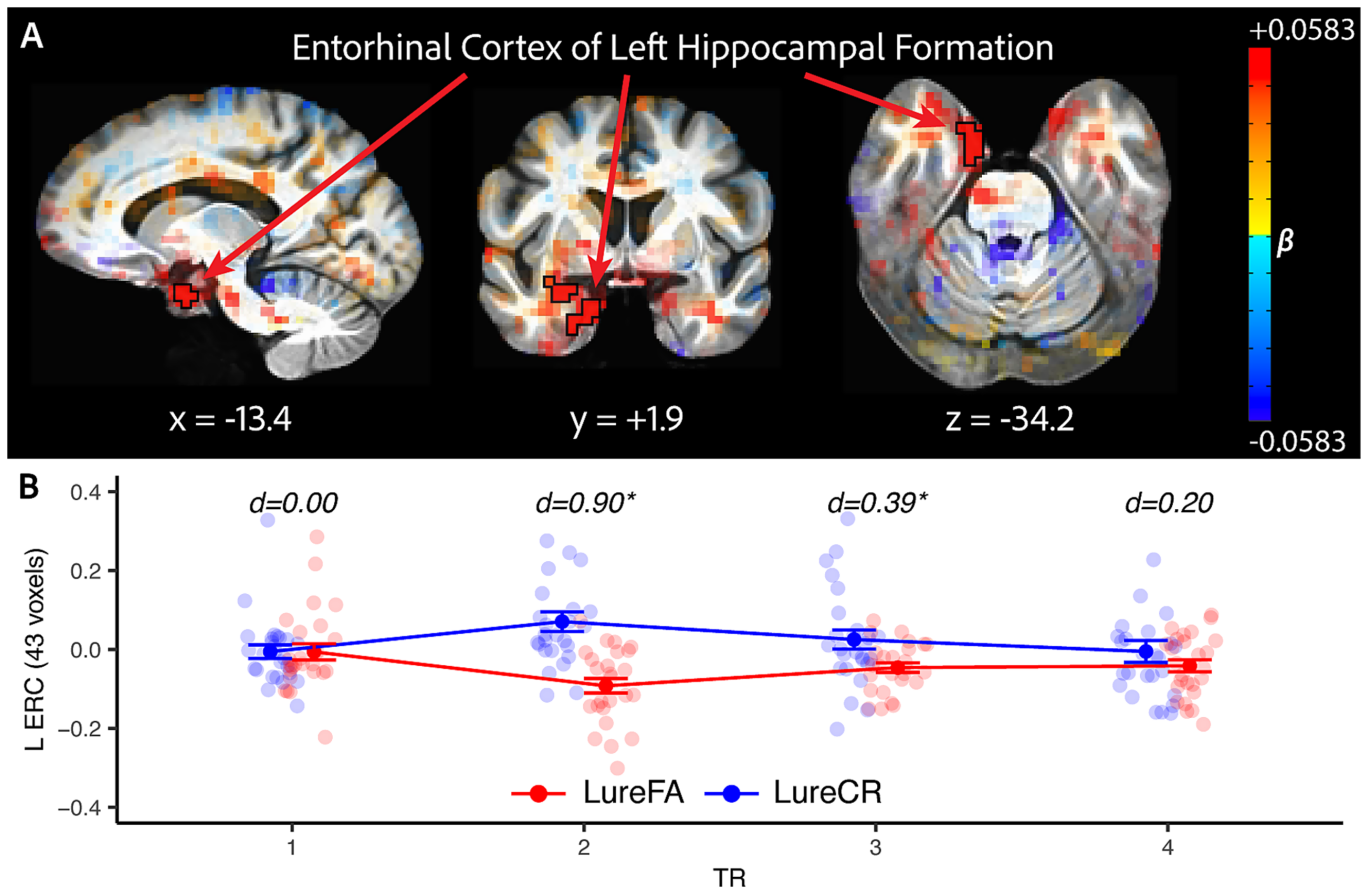
Entorhinal cortex of left hippocampal formation associated with high-fidelity memory from +LureCR-LureFA contrast. The impulse response beta coefficients were compared as +LureCR-LureFA at every time point using linear mixed-effects model analysis, thresholded at *p* < 0.05, and minimum voxel extent = 31. Red color represents positive beta values associated with high-fidelity memory, and blue color represents negative beta values associated with high-fidelity memory. **(A)** Shows a brain-wide statistical parametric map with significant clusters outlined by black lines and non-significant clusters presented as semi-transparent. **(B)** Shows the average “tentzero” impulse response for the respective hippocampal clusters. Cohen’s d is displayed for each comparison, and asterisks denote statistical significance (*p* < 0.05). Coordinates are expressed as MNI coordinates. L ERC, left entorhinal cortex, LureCR (Lure—correct rejection), LureFA (Lure—false alarm), repetition time (TR).

Using linear mixed-effects models (*3dLME*) and a brain mask, the contrast of +LureCR -LureFA was performed to capture the MTL processes associated with hippocampal-dependent high-fidelity LTM retrieval (i.e., mnemonic discrimination). The use of lure (*LureCR* and *LureFA*) trials provides relevant functional correlates of MTL-dependent pattern separation memory processes. Cluster-level correction for multiple comparisons was performed using AFNI 3dClustSim with spatial autocorrelation function parameters estimated from the 3dLME residuals (3dFWHMx). Simulations were conducted using a brain mask in template space (3 mm isotropic resolution), and two-sided thresholding was used to determine the minimum cluster extent required to achieve corrected significance thresholds. The minimum cluster extent thresholds are tabulated in Supplementary Table S1. The statistical maps were voxel-wise thresholded at *p* < 0.01 and minimum voxel extent >31 voxels (837 mm^3^) to capture reliable patterns of activation. One cluster was found in the left entorhinal cortex at this threshold (Figure 2) and was used as a seed region-of-interest (ROI) mask for subsequent beta-series functional connectivity analysis.

### 2.5 Trial-wise beta-series correlation analysis of MTL-lateral cortical functional connectivity

Trial-wise beta-series correlation was performed with the left entorhinal cluster using linear mixed-effect contrast (Gazzaley et al., 2004; Rissman et al., 2004). Each participant’s new design matrix modeled each lure trial’s impulse response (*TENTzero*, 0–10 s in 2 s increments) individually (using the *-stim_times_IM* option of AFNI’s *3dDeconvolve*), and each beta-series coefficient was extracted for two conditions: LureCR and LureFA (via *3dbucket*). The same six motion parameters, third-order polynomial baseline fits, CSF, and white matter nuisance regressors derived from the initial *afni_proc.py* command were included in the regression.

The left entorhinal group-defined ROI mask was back-transformed into individual native anatomical space (via ANTs) to obtain each participant’s correct MTL ROIs and then seed average beta series calculations (*3dmaskave*), which correlated the activation across the time series against cortical gray matter voxels (*3dTcorr1D*). Whole-brain correlation maps were Fisher’s z-transformed and transformed back to the custom ANTs group template (*antsApplyTransforms*) for group analyses.

A paired *t*-test (*3dttest++*) was performed with the contrast “*+LureCR -LureFA*” to observe which cortical regions yielded greater connectivity during correct rejection of lures relative to false alarms (i.e., LureCR versus LureFA, successful mnemonic discrimination). Statistical maps were thresholded at voxel-wise *p* < 0.05 and minimum voxel extent >138 voxels to capture reliable patterns of activation (see Supplementary Table S1 for minimum cluster extent thresholds). The cortical regions were inspected for significance. These broad prefrontal and parietal regions were chosen because of their prior associations with cognitive control processes during LTM retrieval (Barredo et al., 2015; Dulas and Duarte, 2016; King et al., 2015; Wais et al., 2017; Wang et al., 2016). Although the broad clusters corresponded to multiple regions of interest, a separate mask of the significant (*p* < 0.05) subregions, including the inferior frontal gyri, precuneus, and angular gyri, was drawn to measure the effect sizes of these memory processes.

## 3 Results

### 3.1 Behavioral results

Performance was assessed via the accuracy of the old/new responses entered into three stimulus categories: targets (an object presented during the study session), similar lures (the paired object similar to the target but presented only during the test session), and novels (a completely new object presented only during the test session). Table 2 shows the group mean proportions for accuracy, d’, and LDI. The LDI accounts for participants’ application of individual strategies (i.e., liberal to conservative; Macmillan and Creelman, 2005) in solving the memory task by correcting their lure rejection rates with respective false alarm rates for the novel stimuli (Wais et al., 2017).

**Table 2:** Behavioral results.

Behavioral results (mean ± SEM)	Targets		Lures		Novels	
	Hit	Miss	CR	FA	CR	FA
Number of trials	107.4 ± 15.8	31.5 ± 15.7	71.1 ± 18.5	68.0 ± 17.9	48.3 ± 1.7	1.1 ± 1.0
Number of trials used in fMRI modeling	107.3 ± 15.7	31.1 ± 15.7	71.1 ± 18.5	67.5 ± 17.7	48.3 ± 1.7	0.0 ± 0.2
Overall accuracy (mean ± SEM)	0.77 ± 0.02	0.23 ± 0.02	0.51 ± 0.03	0.49 ± 0.03	0.94 ± 0.01	0.06 ± 0.01
d’ (mean ± SEM)	1.11 ± 0.10					
LDI (mean ± SEM)	0.45 ± 0.04					

Group means accuracies for responses to target, lure, and novel images (standard error of the mean [SEM]) are presented, as well as the mean d-prime (d’) and Lure Discrimination Index (LDI). D′ is calculated as z(HitRate)−z(False Alarm) . LDI is calculated as Pr(LureCR)−Pr(NovelFA) . Correct rejection (CR), false alarm (FA), and functional magnetic resonance (fMRI). The number of trials used in fMRI modeling was lower because of rejected trials due to excessive motion.

### 3.2 Univariate contrast results of high-fidelity memory

After correcting for false discoveries, one cluster of 43 voxels was found in the left entorhinal cortex. The most robust difference was observed in the second TR (i.e., 4 s after stimulus presentation) with *t(175)* = 2.60, *p* = 0.0101, Cohen’s *d* = 0.90 (Figure 2B).

### 3.3 High-Fidelity memory functional networks: event-related trial-wise beta-series correlations

**Figure 3:**
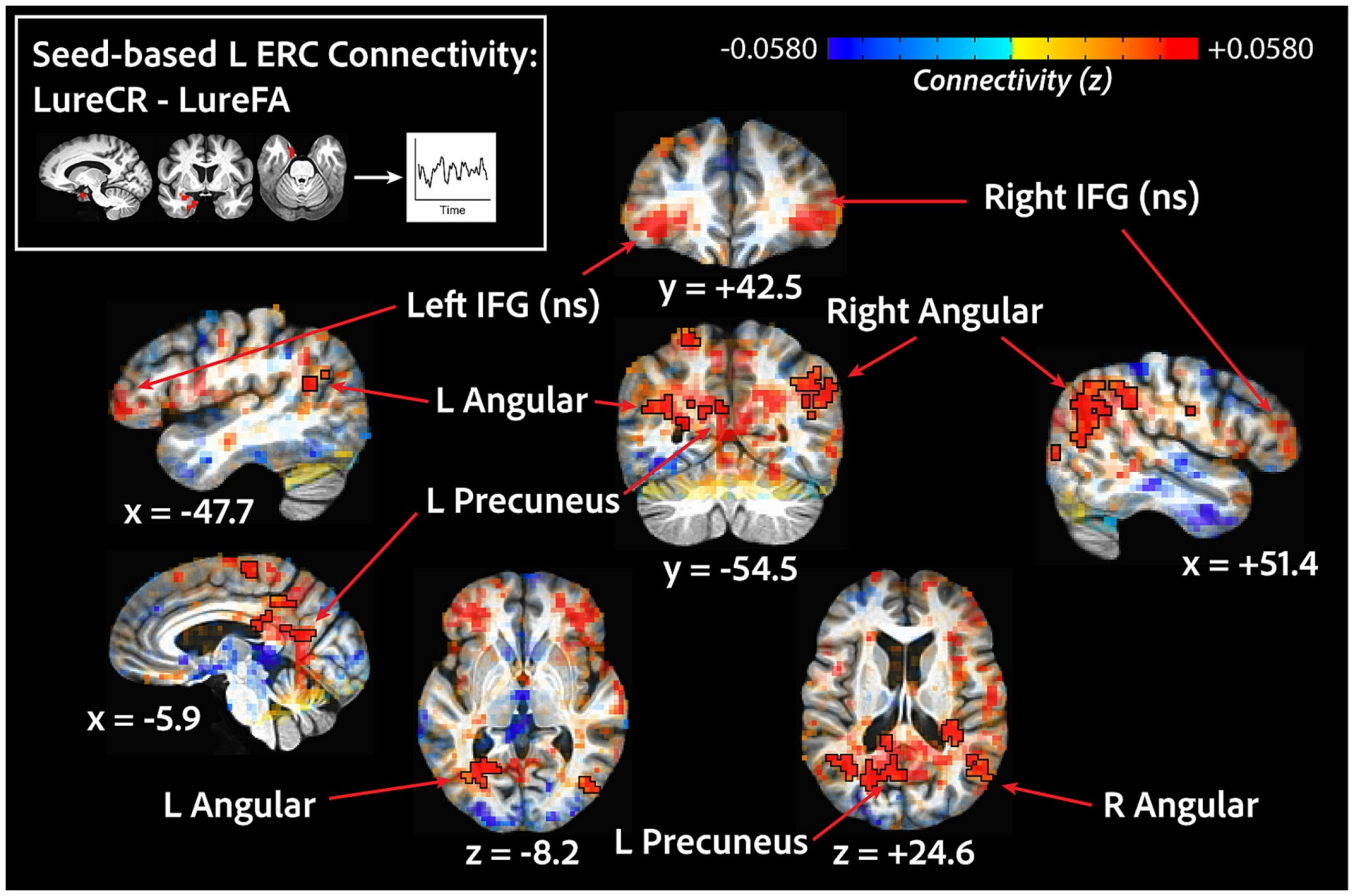
Beta series correlation seeds for a hippocampal formation region in the left entorhinal cortex (L ERC). L ERC-seed-based functional connectivity metrics were z-transformed and contrasted between the LureCR and LureFA trials. The x/y/z coordinates correspond to the right/anterior/superior MNI coordinates. Red color represents positive correlations in functional networks between regions, and blue color represents negative correlations in functional networks between regions. left (L), right (R), inferior frontal gyrus (IFG), not significant (ns).

Beta-series correlations of trial-wise activity showed that the hippocampal formation region in the left entorhinal cortex (L ERC) was functionally connected to broadly distributed cortical regions. L ERC showed greater functional connectivity in association with trials given LureCR responses than those given LureFA responses in the right angular gyrus and left precuneus (Figure 3). The regions of the bilateral inferior frontal gyrus also showed trends toward significance (but did not meet the cluster extent thresholds). Table 2 and Figure 3 isolate the relevant regions and list the significant contributing voxels (thresholded voxel-wise *p* < 0.05).

## 4 Discussion

**Figure 4:**
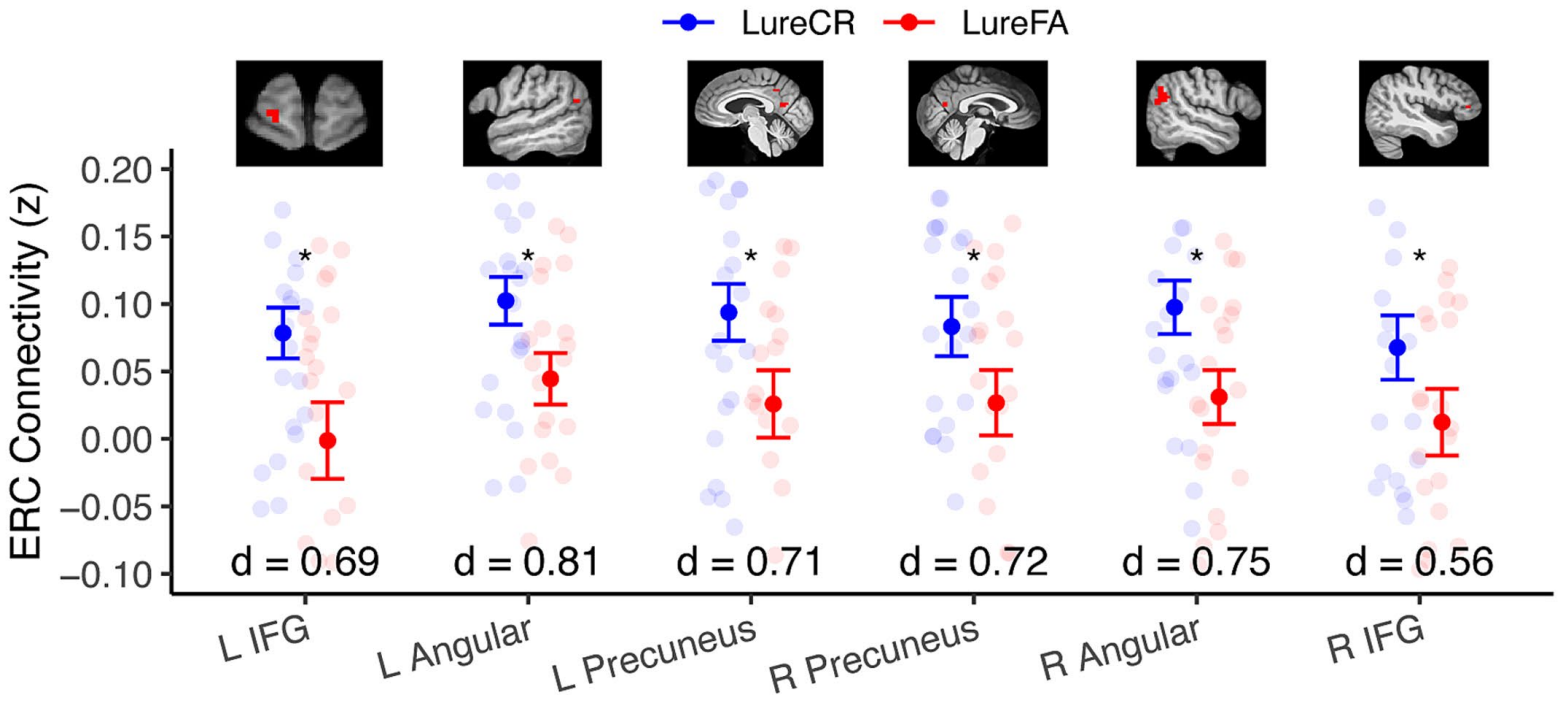
Greater left hippocampal-cortical functional connectivity in association with high-fidelity memory. Voxels belonging to relevant regions and individual-level connectivity estimates were subjected to paired t-tests. Fisher’s z-transformed connectivity to the left entorhinal cortex (ERC) is shown for the LureCR (blue) and LureFA (red) trials. Points and error bars refer to mean ± standard error; asterisks denote false discovery rate-corrected significance from paired t-tests; Cohen’s d is included in the inset; faded points show a scatter distribution of individual values. Correct rejection (CR), false alarm (FA), left (L), right (R), entorhinal cortex (ERC), inferior frontal gyrus (IFG).

We assessed the capability for high-fidelity LTM at retrieval in healthy older adults using a well-established behavioral pattern separation task. Our cohort’s LDI score was 0.45 ± 0.04, significantly below the level of younger adults on the identical task—0.56 ± 0.04 (Wais et al., 2017; Supplementary Table S2)—which was expected based on findings from a substantial body of literature of relevant results (Chalfonte and Johnson, 1996; Hashtroudi and Johnson, 1990; Naveh-Benjamin et al., 2003; Stark and Stark, 2017). We first identified the MTL region supporting high-fidelity memory (Figure 2) and then performed trial-wise beta-series correlation in successful and unsuccessful lure discrimination trials to uncover cortical networks that support high-fidelity memory (Figure 4). These regions (i.e., the right angular gyrus and left precuneus) were found to be more functionally connected during successful lure discrimination. The differential cortical connectivity patterns (particularly the precuneus) suggest age-related differences from previously studied young adult cortical connectivity patterns, which will be discussed further below.

The left entorhinal cortex, a gateway region for signals in and out of the hippocampal formation, appeared to be fundamental for successful lure discrimination (Figure 2). This is in line with previous studies implicating the entorhinal cortex in lure discrimination in young adults (Reagh and Yassa, 2014; Wais et al., 2017), older adults (Adams et al., 2022), and older adults with mild amnestic cognitive impairment (Tran et al., 2022). The entorhinal cortex contains the perforant pathway—a primary pathway through which the hippocampus connects to the cortex—and the integrity of this pathway is related to mnemonic discrimination performance (Bennett and Stark, 2016; Yassa et al., 2011). The entorhinal cortex is among the first affected regions in Alzheimer’s disease (Braak et al., 2008), which further reinforces the importance of the entorhinal cortex in high-fidelity memory.

Trial-wise beta-series correlations over the time series of the memory test identified functional high-fidelity LTM networks (Figure 4) that showed greater connectivity between the MTL and the right angular gyrus and left precuneus, as well as notable trends with the inferior frontal gyrus. These connectivity increases were associated with correctly rejecting similar lures as new (LureCR) relative to false alarms to similar lures (LureFA). Correct rejection of lures in an object recognition task is a critical behavioral outcome operationalized via a behavioral pattern separation task (Stark et al., 2013), which has been associated with hippocampal-dependent LTM processes (Bakker et al., 2008). The angular gyrus and precuneus are both parts of the default mode network, which further corroborates prior resting-state investigations of the brain networks that underlie high-fidelity memory (Wahlheim et al., 2022). Importantly, this pattern defines the recruitment of the MTL–cortical memory networks in healthy older adults. We discuss these cortical regions in subsequent paragraphs.

The present results did not find robust increases in functional connectivity between the MTL and IFG regions in support of high-fidelity memory, in contrast to previous results. The IFG is thought to be important for successful high-fidelity memory retrieval (Preston and Eichenbaum, 2013) and has been associated with the selection of relevant details during LTM retrieval efforts, which resolves interference with competing similar memories (Badre et al., 2005). The IFG and hippocampus are linked by a ventral retrieval pathway (Barredo et al., 2015), whereby cognitive control processes that guide the resolution of interference across memories are connected with pattern separation processes dependent upon the MTL (Bein et al., 2020). The present results show subthreshold clusters of IFG involvement in high-fidelity memory (Figure 4), which may be due to general gray matter shrinkage with age (Taki et al., 2011), leaving fewer voxels to contribute to meeting the cluster extent thresholds. Alternatively, neural dedifferentiation in older adults—characterized by reduced focal selectivity and broader, lower-magnitude recruitment (Koen and Rugg, 2019)—may explain why the present IFG findings suggest notable trends but did not survive thresholding in high-fidelity memory (Figure 4).

The posterior parietal cortex (PPC), which incorporates the angular gyrus, has been implicated in the retrieval of high-fidelity LTM by both younger and older adults (Ciaramelli et al., 2010; Davis et al., 2008, 2012). PPC is also associated with a broader default mode network (DMN) and has been interpreted as a store of memory representations abstracted beyond sensory constraints (Chen et al., 2017; Chen et al., 2022). According to this interpretation, increased angular gyral activity reflects attentional adjustments triggered by MTL activity, as proposed by the attention-to-memory model (Cabeza et al., 2008). Past studies in young adults have associated the left angular gyrus with the vividness of memory recall (Kuhl and Chun, 2014), and stimulation of a hippocampal-PPC network improved the precision of recollection (Nilakantan et al., 2017). Other studies specific to mnemonic discrimination have associated bilateral angular gyri activity and functional connectivity with MTL with high-fidelity LTM in young adults (Stevenson et al., 2020; Wais et al., 2017). The left angular gyrus, unilaterally, has been associated with episodic memory in studies on young adults (Berryhill, 2012; Schoo et al., 2011; Thakral et al., 2017). The results from the present study indicate that connectivity between the left entorhinal cortex and right angular gyrus functions as a network underlying high-fidelity memory in older adults (Figure 4). Our findings suggest that the right angular gyrus in older adults may accumulate contextual information retrieved from memory processes that support high-fidelity memory (King et al., 2015).

Interestingly, our results here show that increased precuneus-entorhinal connectivity is associated with high-fidelity memory retrieval (Figure 4). For the precuneus, task-related activation, in association with subjective recollection (Angel et al., 2013) or source memory retrieval (Meusel et al., 2017), has been shown to increase precuneus contribution to memory accuracy. However, the contributions of the precuneus to episodic or autobiographical memory retrieval are not well understood in older adults. While one study with younger adults implicated precuneus activation in lure discrimination (Pidgeon and Morcom, 2016), it is significant that connectivity with the MTL was not evident in the pattern of distributed functional networks associated with high-fidelity LTM retrieval (Wais et al., 2017, 2018). As the precuneus is thought to evaluate the validity of memory judgments (Ye et al., 2018) and there is an increased likelihood of false beliefs in older adults (Dodson et al., 2007; Stark et al., 2019), we interpret the present MTL-precuneus network as recruitment of supplementary processes that older adults engage in when evaluating the relevance and specificity of details they retrieve in high-fidelity memories. Notably, previous studies have implicated the precuneus as one of the earliest regions to exhibit age-related changes in function (Billette et al., 2022) and structural morphology (Putcha et al., 2011). A study collecting longitudinal assessments of LTM performance and amyloid-beta accumulation found that elevated precuneus activity in association with memory retrieval was also correlated with greater amyloid-beta burden (Fischer et al., 2025), although LTM performance over four years did not change. Thus, our present finding, which shows broad left precuneus-MTL functional connectivity in association with high-fidelity LTM retrieval, provides insight into the LTM networks in healthy older adults in the context of future risk of cognitive decline. Moreover, from the perspective of a behavior-first research framework to examine functional brain networks in older adults (Mooraj et al., 2025), our novel findings elucidate MTL–cortical connectivity arising in association with the retrieval of high-fidelity details in LTM.

Limitations of the present study include the inability to directly compare statistical maps between the old and young cohorts and the lack of a longitudinal comparison across the older group. Our methodology using functionally defined MTL seed regions for high-fidelity retrieval, at its core, cannot provide a direct comparison of older adults’ memory networks against those mapped from a separate cohort of young adults. We used functionally defined MTL seeds to perform trial-wise beta-series correlations, and the young and old cohorts likely used different distributions of MTL regions to support high-fidelity memory. Interpretation of these results may be problematic, as fMRI comparisons between young and adult individuals may be attributed to other age-related effects, such as cardiovascular differences (Tsvetanov et al., 2015), broader variability in activation regions (Kannurpatti et al., 2010), and hemodynamic response function differences (D’Esposito et al., 1999; West et al., 2019). Instead, including longitudinal comparisons would allow for stronger claims regarding aging-related processes (Cabeza et al., 2018). Future studies are needed to further investigate what functional memory network differences exist between older adult cohorts and young adult cohorts or how these memory networks evolve across years in association with aging-related processes.

An additional limitation is that the approach to reduce sampling variability in the number of lure trials for modeling fMRI data necessitated the collapse of responses with a scale of confidence ratings into binary old/new decisions. Each participant contributed differing amounts of lure trials to correct rejection and false alarm conditions—with high performers contributing more LureCR trials and necessarily fewer LureFA trials—the present paradigm reduces this concern by leveraging an optimally moderate difficulty of approximately 50%, such that the number of LureCR and LureFA trials was mostly balanced for most participants (Table 3). Finally, we collapsed across confidence ratings in light of several participants’ exclusive use of “definitely old” or “definitely new” responses—which would make the modeling of confidence and its subsequent relevance to associative memory tenuous given the further reduced sample sizes. For transparency, the number of participant responses to the lure objects is shown in Supplementary Table S3.

**Table 3:** Summary of regions associated with memory discrimination processes.

Analysis	Location	MNI x	MNI y	MNI z	Voxels	*t*	*p*	*d*
3dLME: LureCR (t1)—LureFA (t1)	L ERC	−13.4	+1.9	−34.2	43	5.9	1.4e-8	0.90
Trial-wise beta-series correlation based on left ERC: LureCR–LureFA	L IFG	−19.8	65.6	−3.3	11	3.2	0.005	0.69
	L Angular	−47.7	−54.5	+22.9	23	3.7	0.001	0.81
	L Precuneus	−5.9	−54.8	+23.0	22	3.2	0.004	0.71
	R Precuneus	+2.4	−64.9	+24.6	7	3.3	0.003	0.72
	R Angular	+56.1	−57.0	+30.2	67	3.4	0.002	0.75
	R IFG	50.2	−43.5	−7.2	5	2.6	0.017	0.56

Montreal Neurological Institute (MNI), lure correct rejection (LureCR), lure false alarm (LureFA), entorhinal cortex (ERC), left (L), right (R). Voxels refer to the number of significant (*p* < 0.05) voxels corresponding to the location. T-statistics, *p*-values, and Cohen’s d values are included.

In conclusion, the present study characterized the distributed functional networks underlying the retrieval of high-fidelity LTM in healthy older adults using a mnemonic discrimination task. Entorhinal activation in the hippocampal formation and entorhinal-cortical connectivity, particularly with the precuneus and angular gyrus, are salient characteristics of networks associated with high-fidelity LTM in older adults. This extends the literature on the brain regions implicated in high-fidelity memory in older adults.

## Data Availability

The datasets presented in this study can be found in online repositories. The names of the repository/repositories and accession number(s) can be found at: doi: 10.6084/m9.figshare.28692338.
