# Supplementary material for: Hippocampal formation–cortical high-fidelity memory networks in healthy older adults during the mnemonic discrimination task
Source: Front Aging Neurosci. 2026 Jul 8;18:1788092. doi: 10.3389/fnagi.2026.1788092 (PMC13388291; doi:10.3389/fnagi.2026.1788092)
Supplement: Supplementary file 1 [file Data_Sheet_1.PDF]

Supplementary Materials

Supplementary Table 1 – Minimum cluster extent thresholds generated by AFNI 3dClustSim for two-sided voxel-wise statistical thresholds. Cluster sizes indicate the minimum number of contiguous voxels required to achieve the specified family-wise error corrected p-value.

```
# 3dClustSim -mask mask_3mm.nii.gz -acf 0.566967 3.05552 6.52449
# 2 sided thresholding
# Grid: 53x80x85 3.00x3.00x3.00 mm3 (45147 voxels in mask)
```

p-threshold	0.10	0.05	0.02	0.01
0.05	118.5	137.6	164.3	181.0
0.02	45.4	52.5	62.1	69.8
0.01	27	31	36.8	41.2
0.005	17.4	20.3	23.9	27.3
0.002	10.6	12.4	15.0	16.9
0.001	7.7	9.0	10.9	12.3
0.0005	5.7	6.7	8.2	9.4
0.0002	4.0	4.8	5.9	6.7
0.0001	3.1	3.8	4.7	5.4

Supplementary Table 2 – Mnemonic Discrimination Task Behavioral Scores Comparing Between Young Adult Cohort (from Wais et al., 2017) and the present Older Adult Cohort. Abbreviations: correct rejection (CR), false alarm (FA), d-prime (d'), lure discrimination index (LDI), degrees of freedom (df).

Parameter	Young Adults (N = 20)	Older Adults (N = 22)	*t-value (df), p-value*
Target (Hit)	0.81 ± 0.02	0.77 ± 0.02	*t = 1.22 (39.7), p = 0.23*
Target (Miss)	0.19 ± 0.02	0.23 ± 0.02	*t = -1.22 (39.7), p = 0.23*
Lure (CR)	0.63 ± 0.03	0.51 ± 0.03	*t = 3.10 (39.9), p = 0.004*
Lure (FA)	0.37 ± 0.03	0.49 ± 0.03	*t = -3.10 (39.9), p = 0.004*
Novel (CR)	0.91 ± 0.01	0.94 ± 0.01	*t = -0.50 (39.6), p = 0.62*
Novel (FA)	0.07 ± 0.01	0.06 ± 0.01	*t = 0.50 (39.6), p = 0.62*
d'	1.54 ± 0.10	1.11 ± 0.10	*t = 3.01 (39.8), p = 0.005*
LDI	0.56 ± 0.04	0.45 ± 0.04	*t = 2.30 (39.7), p = 0.027*

Supplementary Table 3 – Confidence in Ratings. The number of responses in each category to lure objects.

Participant	Lure "Definitely Old"	Lure "Maybe Old"	Lure "Maybe New"	Lure "Definitely New"
1	9	47	65	16
2	25	58	26	26
3	10	55	47	25
4	47	24	6	58

5	28	38	11	61
6	33	22	18	66
7	36	17	12	25
8	34	13	25	64
9	37	46	37	16
10	37	38	17	37
11	52	5	5	66
12	17	33	26	62
13	65	0	0	71
14	60	2	4	71
15	97	0	0	41
16	60	0	0	71
17	69	0	2	52
18	47	27	39	22
19	22	4	8	101
20	21	17	15	84
21	32	31	31	46
22	68	29	4	32

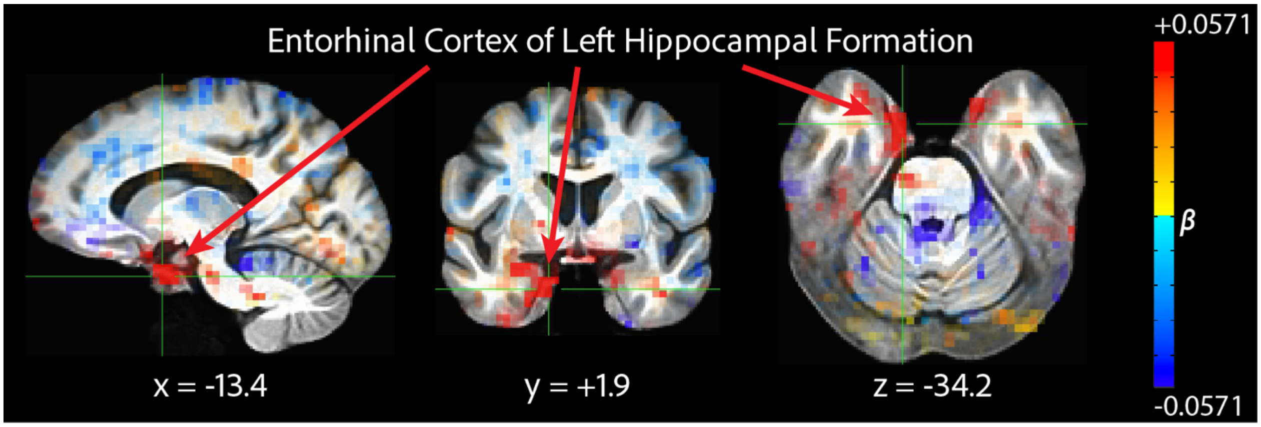

Supplementary Figure S1 – Linear Mixed Effect +LureCR -LureFA Contrast Using Only "Definitely" Responses. Only high confidence trials (e.g., "definitely new" or "definitely old") are included in modeling this. Red colors represent positive beta values, and blue colors represent negative beta values associated with high-fidelity memory.
